# Characterization of the aqueous humor microbiome in Posner–Schlossman syndrome: an exploratory metagenomic sequencing study

**DOI:** 10.3389/fmed.2026.1780981

**Published:** 2026-04-01

**Authors:** Weijia Zhang, Ke Zhang, Yanfeng Liao, Zhen Yang, Ziyao Xia, Xianghan Ke, Di Zhang, Jing Chen, Hongling Wu, Ying Hong, Huaizhou Wang, Ziyuan Liu, Lingge Suo, Yu Zhang, Chun Zhang

**Affiliations:** 1 Beijing Tsinghua Changgung Hospital Eye Center, School of Clinical Medicine, Tsinghua Medicine, Tsinghua University, Beijing, China; 2 Beijing Visual Science and Translational Eye Research Institute (BERI), Beijing, China; 3 Department of Ophthalmology, Peking University Third Hospital, Beijing, China; 4 Department of Ophthalmology, Sinopharm Chongqing Yangtze Shipping Hospital, Chongqing, China; 5 Hangzhou Matridx Biotechnology Co., Ltd., Hangzhou, China

**Keywords:** aqueous humor, exploratory study, metagenomic next-generation sequencing, ocular microbiota, Posner–Schlossman syndrome

## Abstract

**Objective:**

This study aims to characterize the aqueous humor (AH) microbiome in Posner–Schlossman syndrome (PSS) patients and evaluate its potential as a diagnostic and therapeutic target.

**Methods:**

Metagenomic next-generation sequencing (mNGS) was performed on 59 AH samples from patients diagnosed with PSS (*n* = 28) and myopia patients who underwent intraocular lens (ICL) implantation (*n* = 31). Taxonomic profiling and diversity analyses were conducted to characterize the microbial communities. Interactions among microbial community members were evaluated using correlation analyses.

**Results:**

Key findings revealed that intraocular microbiomes existed in both normal and diseased eyes; however, PSS patients exhibited lower microbial diversity (Shannon index, *p* = 0.066; Simpson index, *p* = 0.065) and distinct community structures (PERMANOVA, *p* = 0.05). Disease-specific microbial signatures were identified: *Paeniglutamicibacter* was uniquely enriched in the PSS group, whereas *Escherichia coli* dominated in the ICL group. Moreover, ecological network analysis demonstrated contrasting interaction patterns. The microbiomes in the PSS group formed stable, tightly connected networks with balanced positive/negative correlations, whereas those in the ICL group exhibited antagonistic relationships, suggesting competitive exclusion. These results challenge the traditional view of ocular sterility and reveal dynamic microbiome shifts associated with PSS pathogenesis. The enrichment of *Paeniglutamicibacter* in PSS may represent an associated microbial signature that could potentially reflect compensatory responses to chronic inflammation, although experimental validation is needed to confirm this hypothesis.

**Conclusion:**

Our study provides preliminary evidence supporting the concept of intraocular microbiome dysbiosis in PSS, which requires validation in future studies. These findings suggest that potential microbial biomarkers warrant further investigation for their diagnostic and therapeutic implications.

## Introduction

Posner–Schlossman syndrome (PSS) was first described in 1948 as a disease characterized by recurrent acute episodes of mild, unilateral, non-granulomatous anterior uveitis accompanied by significantly elevated intraocular pressure (IOP) (1, 2). The pathogenesis of PSS remains unclear; however, the majority of studies suggest that it is associated with cytomegalovirus (CMV) infection (3–5). Currently, treatments for PSS primarily focus on antiglaucoma drugs or surgery to reduce IOP, antiviral drugs, and the topical use of corticosteroids to control inflammation (6, 7). However, in many cases, the above treatments fail to prevent the recurrence of PSS and may even lead to corticosteroid dependence, steroid-induced high IOP, or concurrent cataracts. Therefore, a deeper understanding of the pathophysiological mechanisms of PSS is crucial for developing more effective treatments.

In recent years, the study of microbiomes has attracted extensive attention. The human body hosts a large number of microorganisms, which form a commensal, symbiotic, and pathogenic community known collectively as the human microbiome (8). The microbial community plays an important role in maintaining immune balance, participating in metabolism, and defending against pathogens (9, 10). Due to the closed anatomical structure of the eye and the protection provided by the blood–retina barrier, the ocular environment is traditionally considered sterile unless it is invaded by pathogens through unnatural means (11). The role of ocular microorganisms has been increasingly recognized. Recent studies have shown the presence of microorganisms in the eyes and have linked the microbial community in the human aqueous humor to glaucoma (12, 13). Therefore, as a special form of glaucoma, PSS offers a new model for understanding the role of the ocular microbiome in pathological mechanisms.

Metagenomic next-generation sequencing (mNGS) can be used to identify microbial diversity and functional potential at the species level and to determine microbiota composition. It holds promise for advancing our understanding of microbial populations in clinical samples (14). As an advanced technology, mNGS can comprehensively analyze the diversity and functional potential of the microbial community, particularly for the identification of genotypes, species-level classification, elucidation of metabolic pathways, and determination of microbiota composition, and overcome the limitations of traditional analysis (14–16). mNGS has become a promising technology for detecting microbial populations, especially in samples with low microbial abundance (17). The use of mNGS is rapidly transitioning from research laboratories to clinical environments (14, 16, 17).

Therefore, this study aimed to compare the microbiome characteristics of aqueous humor (AH) in PSS patients with myopia who underwent intraocular lens (ICL) implantation surgery with those of controls. We demonstrate an association between changes in the microbiome and the pathogenesis of PSS. Our findings provide a new basis for the early diagnosis and personalized treatment of PSS, deepen our understanding of the role of the microbiome in ophthalmic diseases, and suggest the existence of an ocular microbiome in humans that interacts with the immune microenvironment. This study has clinical significance and will help promote further development of microbiome research in ophthalmology.

## Methods

### Subject recruitment

This study included 59 AH samples, comprising 28 samples from patients with PSS (PSS group) and 31 samples from myopia patients who underwent ICL implantation, serving as healthy controls (ICL group).

The study participants were recruited according to the following criteria: the inclusion criteria for the PSS group were as follows: (1) age 10–80 years; (2) definite diagnosis of PSS based on characteristic clinical features, including recurrent attacks of unilateral elevated IOP with mild non-granulomatous anterior uveitis, attacks lasting hours to weeks, corneal edema, open angle, keratic precipitates, minimal cells, and flare during attacks, and normal IOP and optic disc between attacks; and (3) ability to understand and provide written informed consent. The exclusion criteria were as follows: (1) use of glucocorticoids within 3 months before sample collection; (2) elevated IOP from any other known cause; (3) presence or history of eye diseases other than refractive errors (for controls) or PSS (for cases); (4) history of ocular trauma, infectious uveitis, other immune-related uveitis, or comorbid primary open-angle glaucoma; (5) history of ocular surgery, including anterior chamber paracentesis; (6) history of systemic diseases, including diabetes, hypertension, heart disease, inflammatory bowel disease, cancer, or arthritis; (7) history of immune-related systemic diseases or concurrent infections; and (8) pregnancy or lactation.

De-identified metadata for all 59 samples, including age, sex, diagnosis, eye laterality, IOP, glaucoma medications, preoperative antibiotic exposure, and sampling date, are provided in Supplementary Table S1 to support reproducibility and facilitate future meta-analyses.

### Ethics statement

This study adhered to the tenets of the Declaration of Helsinki. The studies involving human participants were reviewed and approved by the Peking University Third Hospital Medical Science Research Ethics Committee (M2023710) and registered under the Chinese Clinical Trials Registry (ChiCTR2400090540). All samples were obtained with patients’ consent. The patients/participants provided written informed consent for their participation in this study.

### Collection of aqueous humor samples

The AH samples in the PSS group were collected as follows: A topical antimicrobial drug (0.5% levofloxacin eye drops; Cravit, Santen Pharmaceutical Co., Japan) was administered 5 times every 10 min in the eye before surgery. Patients received conjunctival sac irrigation with 0.9% sodium chloride solution at least twice. Following disinfection and draping, 5% povidone iodine (PVI) was applied to the eye for 30 s. The conjunctival sac was then irrigated with 0.9% sodium chloride solution at least three times. After topical anesthesia with 0.5% Alcaine (administered at least three times), an anterior chamber incision was made using a 1-mL disposable insulin injection needle at the 2 or 11 o’clock position of the limbus, and AH was sampled before any other procedures were initiated. Immediately after the collection, the AH samples were transferred to sterile Eppendorf tubes and stored at −80 °C before mNGS examination.

The AH samples in the ICL group were collected as follows: A topical antimicrobial drug, 0.5% levofloxacin eye drops (Cravit, Santen Pharmaceutical Co., Japan), was administered four times a day for at least 3 days before ICL surgery. On the day of surgery, patients received conjunctival sac irrigation using 0.9% sodium chloride solution at least twice and mydriasis using compound tropicamide eye drops. Following disinfection and draping, 5% PVI was applied to the eye for 30 s. The conjunctival sac was then irrigated with 0.9% sodium chloride solution at least three times. After topical anesthesia with 0.5% Alcaine (administered at least three times), an auxiliary incision was made using a 1.5-mm stab knife (Alcon, USA) at the 2 o’clock position of the limbus. The AH was sampled via the auxiliary incision using a 1-mL sterile syringe before any other procedures were initiated. Immediately after collection, AH samples were transferred to sterile Eppendorf tubes and stored at −80 °C before mNGS examination.

### Contamination control measures

To rigorously account for potential environmental and reagent-derived contamination in our low-biomass metagenomic analysis, we included two types of negative controls processed in parallel with the patient samples. Specifically, we collected (1) conjunctival sac irrigation fluid samples after routine povidone–iodine disinfection (*n* = 5) to monitor the surgical field background and (2) balanced saline solution (BSS) samples (*n* = 5) from unopened vials used in the surgical procedures to monitor reagent contamination. These controls underwent the same DNA extraction, library preparation, and sequencing protocols as the patients’ aqueous humor samples.

### DNA extraction and metagenomic sequencing

A frozen aliquot (at least 200 mg) of each AH sample was processed using the Matridx Biological DNA Extraction Kit (Hangzhou Matridx Biotechnology Co., Ltd., China), and an automatic DNA extraction instrument, AutoPure20, was used for DNA extraction according to the manufacturer’s instructions. A DNA library was constructed using the Metagenomic DNA Library Building Kit (reversible terminal termination sequencing method) from Matridx Biotechnology. The library was purified and recycled using a Matridx Biotechnology Purification Kit. The constructed library was quantified by quantitative polymerase chain reaction (qPCR) using a library quantification kit and a real-time PCR system. Based on the quantification results, the samples were pooled for sequencing on the Illumina NextSeq 500 platform, generating an average of 23 million reads per library.

### Sequencing data processing and bioinformatics analysis

Raw sequencing data were initially processed using fastp (default parameters) for quality control to remove adapter sequences and low-quality reads. Subsequently, Bowtie2 (default parameters) was used to align the clean reads to the human reference genome (hg38). Aligned reads were excluded as potential host contamination, and the remaining data were used for downstream analyses.

Taxonomic profiling was performed using Kraken2 and a commercial database. This database integrates the RefSeq complete genome database and the NCBI non-redundant nucleotide sequence database (NCBI nt). To ensure annotation accuracy, the public database was refined using data and validation results from over 500,000 clinical infection samples accumulated by Matridx Corporation, ultimately generating a customized, calibrated database containing more than 30,000 species-level labels.

Species co-occurrence networks were constructed to investigate co-occurrence patterns within microbial communities. Microbial species present in at least 30% of the samples were selected to reduce data sparsity. Subsequently, based on the relative abundance at the species level, Spearman’s rank correlation coefficients (*r*) and their significance (*p*-values) between microbes were calculated. Network construction retained only those correlation pairs with strong correlations and high statistical significance (|*r*| > 0.5 and *p* < 0.001). Cytoscape software was used for network visualization and topological property analysis.

### Statistical analysis

Statistical analyses were conducted using Statistical Package for Social Sciences (SPSS v27.0; IBM, Chicago, IL, USA) and R (v3.6.0) software. Categorical variables (sex, laterality, and age group) were analyzed using the Fisher exact test or the unpaired *t*-test, as appropriate. The Mann–Whitney *U*-test was applied to IOP, glaucoma medications, cup-to-disc ratio (C/D), and best-corrected visual acuity (BCVA). Alpha diversity was assessed using the Shannon and Simpson indices, whereas beta diversity was visualized through principal coordinate analysis (PCoA) using the dudi.pco function in the ade4 package. ANOSIM was performed with the Vegan package in R. Two-sided Wilcoxon rank-sum tests were performed to compare Shannon diversity and Bray–Curtis dissimilarity between groups. Boxplots were used to illustrate the medians (central lines), first and third quartiles (box edges), and 1.5 × interquartile ranges (whiskers).

The diagnostic performance of mNGS was evaluated using the area under the curve (AUC) of the receiver operating characteristic (ROC) curve. The sensitivity and specificity of the detection method were analyzed as previously described (18). Differential feature identification was performed using the linear discriminant analysis effect size (LEfSe) algorithm, which integrates standard statistical significance tests with additional assessments of biological consistency and effect relevance,^1^ applying thresholds of log10 LDA score ≥4 and *p*-value of ≤0.05. Data were presented as the mean ± standard error (SE). Spearman’s correlation coefficients were computed in R and visualized as a network using Cytoscape.

To evaluate the discriminatory performance of microbial features as potential biomarkers distinguishing PSS from ICL, we performed random forest classification using the relative abundance profiles of all microbial species. A five-step learning curve analysis was conducted with training sample sizes of 9, 18, 28, 37, and 47 to assess the relationship between sample size and model accuracy. Subsequently, a 5-fold cross-validation ROC analysis was performed using the full dataset to calculate the mean area under the curve (AUC) and its range. All random forest analyses were implemented using the randomForest package in R (version 4.0.3) with default parameters.

## Results

### Demographic information of the enrolled patients

For this study, a total of 59 AH samples, including 28 eyes from 27 individuals in the PSS group and 31 eyes from 23 individuals in the ICL group, were collected. The clinical information obtained, including sex, left and right eyes, BCVA, IOP, glaucoma medications, and C/D, is shown in Supplementary Table S2. We observed that a greater proportion of the PSS group were male individuals, older, had worse BCVA, higher IOP, and larger C/D. No significant difference was observed in the number of left and right eyes among the samples between the two groups.

### Assessment of background contamination

To ensure the authenticity of the microbial communities identified in the aqueous humor, we sequenced two types of negative controls in parallel: conjunctival sac irrigation fluid and balanced saline solution (BSS). Metagenomic analysis of these controls revealed a low-biomass profile, with only two bacterial genera, GGB7511 and Aquabacterium, detected in some of the samples (Supplementary Figure S1). Reassuringly, neither of these genera constituted a dominant member (e.g., the top 10 abundant genera) in any of the patient or control aqueous humor samples. This finding indicates that the predominant microbial signals reported below are unlikely to stem from procedural or reagent contamination and likely represent true biological signatures present in the aqueous humor.

### Differences in microbial diversity under different conditions

Next-generation sequencing (NGS) was performed on the collected AH samples and negative controls, generating over 23 million clean reads per sample. The results of metagenomic data analysis indicated that only GGB7511 and *Aquabacterium* were found in some of the negative controls. In the ICL and PSS aqueous humor samples, these two bacteria were not dominant (Supplementary Table S3).

On average, approximately 255,445 out of every million clean reads can be identified as microbial by the Kraken2 program in AH samples. After data normalization, microbial diversity indices and Bray–Curtis distances were calculated for each sample. Principal coordinate analysis (PCoA) was conducted based on these distances, revealing the spatial distributions of all samples. The first principal coordinate (PCoA1) accounted for 54.21% of the variation, whereas the second principal coordinate (PCoA2) explained 19.63%.

**Figure 1:**
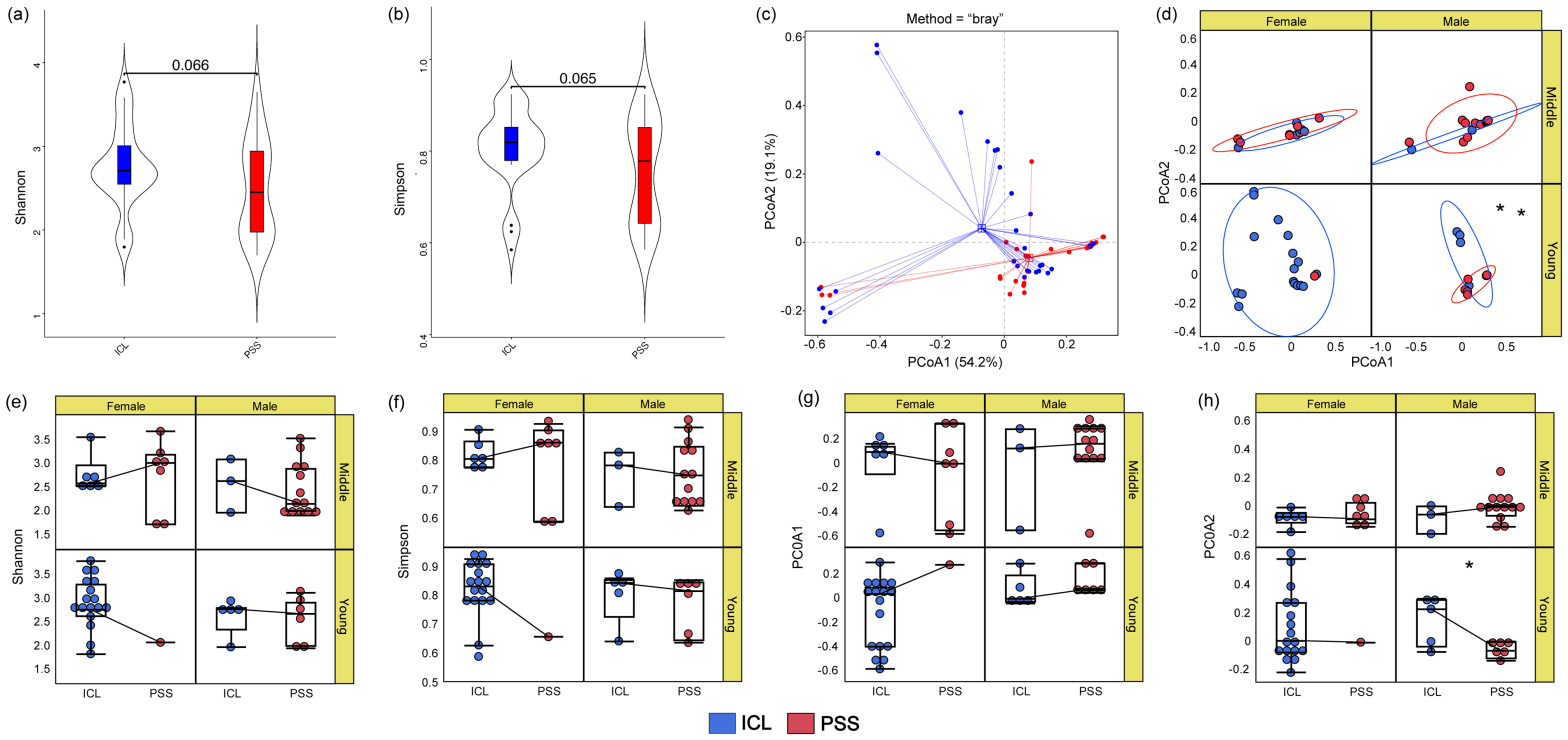
Microbial diversity analysis of aqueous humor metagenomic sequencing data from the PSS and ICL groups. The PSS samples are indicated by red dots and lines, whereas the ICL samples are indicated by blue dots and lines. **(a,b,e,f)** Differences between the Shannon and Simpson diversity indices; **(c)** PCoA results for all samples; **(d)** PCoA results stratified by age and sex; **(g,h)** differences in the PCoA1 and PCoA2 axes under the influence of age and sex factors.

Microbial diversity indices were compared between the PSS and ICL groups, indicating a lower overall diversity in the PSS group. Using the Wilcoxon test, the *p*-values for the Shannon and Simpson indices were 0.066 and 0.065, respectively, which were not significantly different between the two groups (Figures 1a,b). For the PCoA components, the ADONIS (permutational multivariate analysis of variance) and ANOSIM (analysis of similarities) tests were performed, yielding *p*-values of 0.019 and 0.029, respectively. These results indicated that there was a significant difference in the PCoA distribution between the ICL and PSS groups (Figure 1c). Further analysis considered the potential influence of sex and age. The samples were categorized into two age groups— ≤ 32 years and >32 years—based on the median sample distribution.

The Wilcoxon rank-sum test was used to perform subgroup analysis under specific conditions. Owing to the reduction in sample size, neither the Simpson nor Shannon indices passed the significance test. Significant differences were observed only in the composition of PCoA2 among younger male subgroups (Figures 1d–h).

We further conducted PERMANOVA (adonis) analyses incorporating age and sex as covariates. After adjusting for age and sex, the microbial community composition still showed significant differences between the PSS and ICL groups (PERMANOVA: *R*^2^ = 0.112, *p* = 0.023). Age alone explained 4.8% of the variance (*p* = 0.184); sex explained 3.1% (*p* = 0.342), whereas disease status remained the primary factor driving community differences.

### Microbial composition differences between the PSS and ICL groups

**Figure 2:**
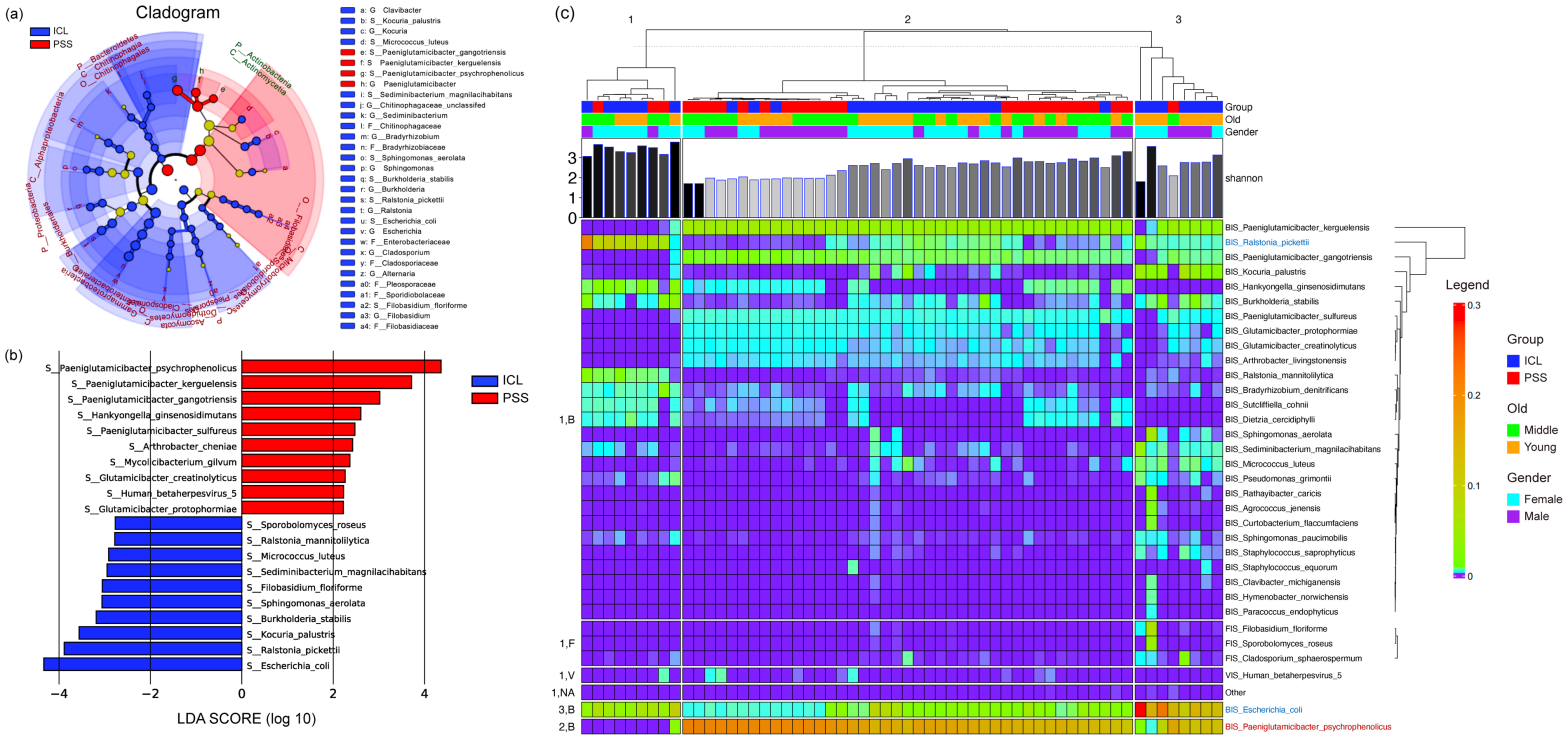
Differential microbial compositions between the PSS and ICL groups identified using LEfSe analysis. **(a)** Cladogram depicting the taxonomic hierarchy of the differentially abundant microbial taxa (LDA > 3). The ICL group exhibited greater diversity, whereas the PSS group exhibited greater dominance of *Paeniglutamicibacter*. **(b)** The top 10 differentially abundant microbes at the species level are ranked by LDA scores. The vertical axis represents the LDA score, with positive values indicating enrichment in the PSS group and negative values indicating enrichment in the ICL group. **(c)** Heatmap of differentially abundant species (LDA > 2). The differentially abundant microorganisms were categorized into three distinct clusters (*Ralstonia, Escherichia, and Paeniglutamicibacter*), and the dominant taxon was significantly enriched among younger individuals in the ICL group.

We employed LEfSe analysis to identify differentially abundant microorganisms between the PSS and ICL groups (Figures 2a,b). At the hierarchical cladogram level, the distributions of species in the ICL group exhibited greater diversity. Microorganisms enriched in the ICL group were distributed across multiple phyla, including Bacteroidetes, Proteobacteria, Ascomycota, and Alphaproteobacteria. Based on the LDA scores, *Escherichia coli* was identified as the most representative species in the ICL group at the species level. In contrast, microbial differences in the PSS group were primarily concentrated in the phyla Actinobacteria and Bacteroidetes, with several species from the genus *Paeniglutamicibacter*—such as *Paeniglutamicibacter psychrophenolicus*, *Paeniglutamicibacter kerguelensis*, and *Paeniglutamicibacter gangotriensis*—showing greater abundance.

### Correlation between *Paeniglutamicibacter* abundance and microbial diversity and clinical parameters

To further explore the ecological and clinical relevance of the disease-enriched taxon, we performed correlation analyses between the abundance of *Paeniglutamicibacter psychrophenolicus* and both microbial diversity indices and key clinical parameters.

**Figure 3:**
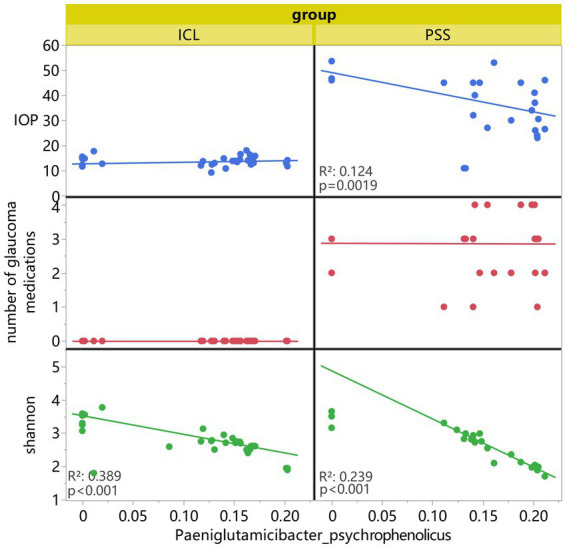
Fitted correlations between the four variables. The *X*-axis represents the abundance of *Paeniglutamicibacter* spp., and the *Y*-axes correspond to intraocular pressure (IOP), number of glaucoma medications, and the Shannon index, respectively.

Notably, we observed a significant negative correlation between *Paeniglutamicibacter psychrophenolicus* abundance and the Shannon diversity index in both the PSS and ICL groups (Figure 3), indicating that the increased dominance of this taxon is associated with reduced overall microbial community complexity. This association persisted across both the disease and control groups, suggesting that *Paeniglutamicibacter* may play a role in shaping the intraocular microbial ecosystem independent of disease status.

When examining clinical parameters, we identified a significant negative correlation between *Paeniglutamicibacter psychrophenolicus* abundance and IOP, specifically in the PSS group, whereas no such association was observed in the ICL group. In contrast, no significant correlation was found between *Paeniglutamicibacter psychrophenolicus* abundance and the number of glaucoma medications in either group, suggesting that the observed microbial-clinical associations are not confounded by treatment intensity.

### Characteristics of the three types of aqueous humor (AH) microbiota

**Figure 4:**
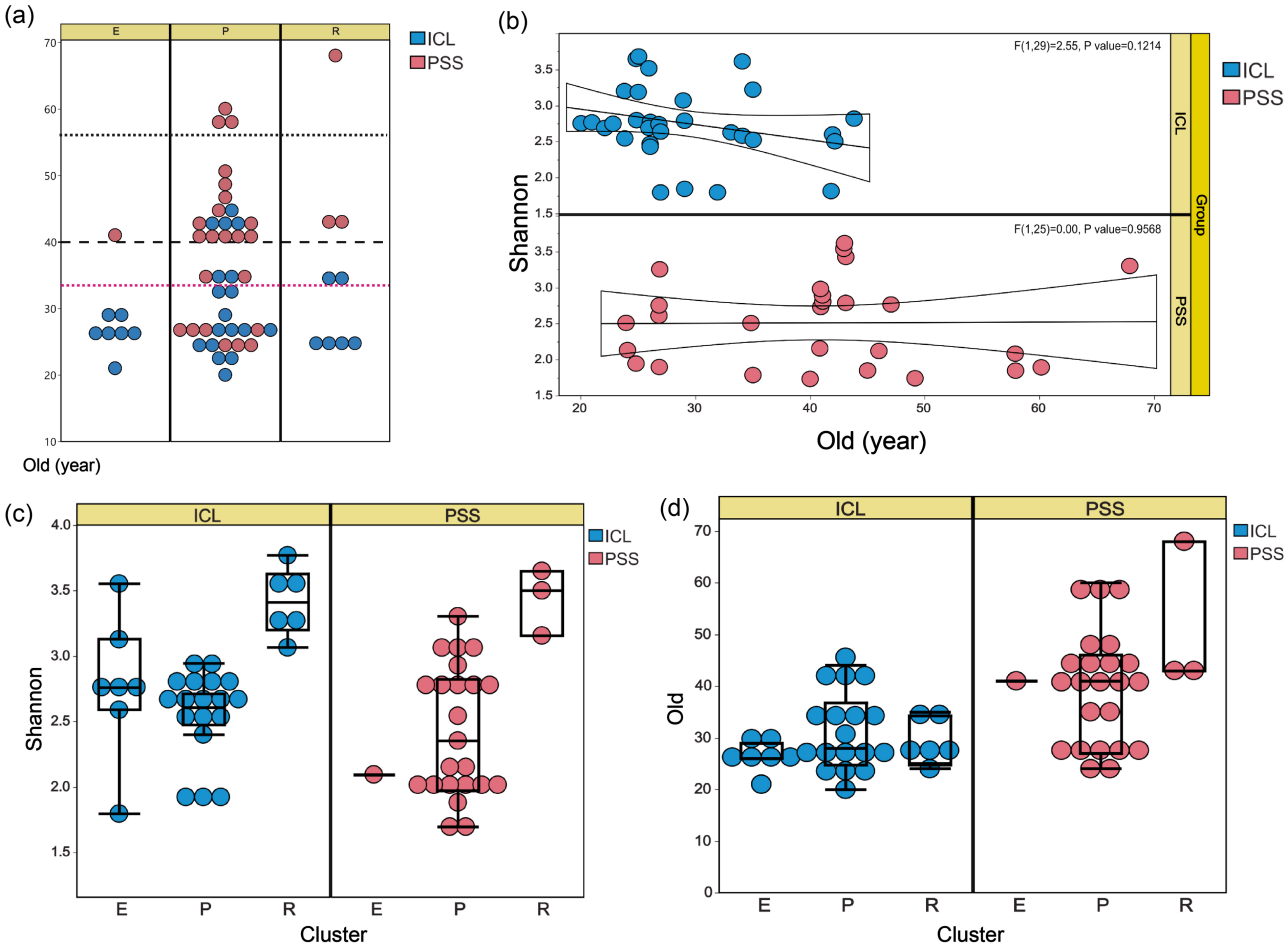
Microbial diversity and age distribution across aqueous humor microbiome clusters. **(a)** Age stratification strategies. The red dashed line indicates the grouping scheme adopted in this study, dividing the subjects into two equal-sized age groups of 33 years. The dashed black line represents an alternative grouping scheme that was not used. Both stratification methods produced consistent results. **(b)** Linear regression analysis between age and Shannon diversity indices. **(c)** Shannon diversity indices among the three microbiome cluster types. **(d)** Age distribution across the three microbiome cluster types.

Using hclust and *k*-means for unsupervised clustering, all samples were divided into three categories based on the composition of differential microorganisms (LDA > 2). *Escherichia coli*, *Ralstonia pickettii*, and *Paeniglutamicibacter psychrophenolicus* served as the most abundant representative microorganisms for each cluster, ranking first and second in the LDA test for the ICL group and first in the PSS group, respectively. The statistical analysis of the characteristics of the three microbial clusters revealed that the cluster represented by *Ralstonia pickettii* exhibited a significantly greater Shannon diversity index compared to the other two clusters, as determined by the Dunn test. Moreover, using the Fisher exact test, the cluster represented by *Escherichia coli* showed enrichment patterns in both the young and ICL groups (Figure 4a). In contrast, the cluster represented by *Paeniglutamicibacter psychrophenolicus* displayed the opposite pattern, with significant enrichment in the middle and PSS groups (Supplementary Table S4). In addition, we investigated the relationship between age and microbial diversity and detected no significant association between age and the Shannon diversity index (Figure 4b). However, there was a significant correlation between microbial community clusters—particularly the *Ralstonia pickettii* cluster—and microbial diversity (Figure 4c). While the age composition in the ICL group did not differ significantly across clusters, the diversity within the *Ralstonia pickettii* cluster was significantly greater than that in the other clusters (Figure 4d).

### Interaction networks among microbial communities in the PSS and ICL groups

**Figure 5:**
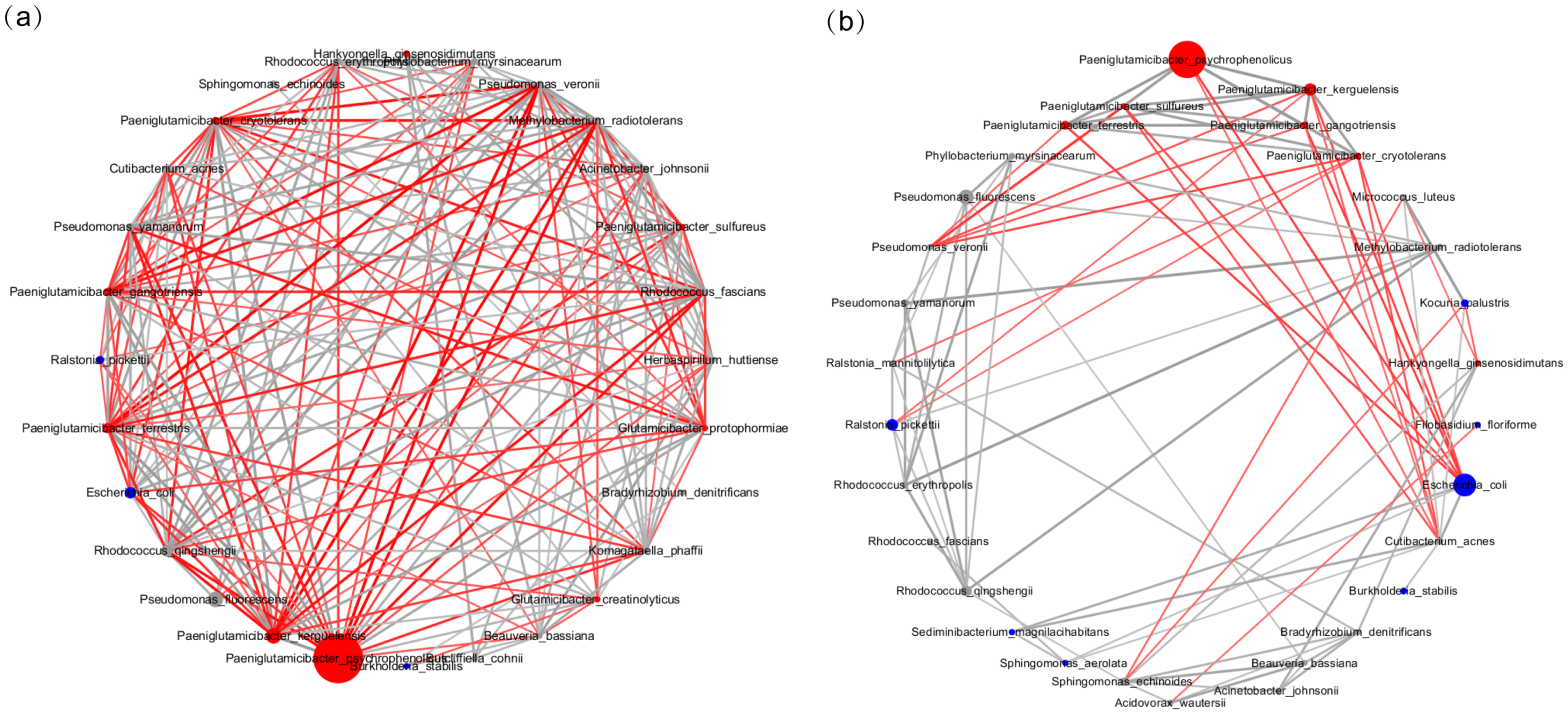
Network of correlations among the top microbial species within the PSS and ICL groups. **(a)** Correlation network within the PSS group. **(b)** Correlation network within the ICL group. The network illustrates the correlation patterns among the top species (the top 30, subject to confirmation) in the PSS and ICL groups. Red edges indicate negative correlations, whereas gray edges indicate positive correlations. Only correlations with *p* < 0.01 are displayed. Red nodes represent microbial species enriched in the PSS group, and blue nodes represent those enriched in the ICL group. The size of each node is proportional to its average relative abundance.

To further elucidate the interactions among microbial community members, we calculated Spearman correlations between microbial taxa within the PSS and ICL samples. Relationships with a *p*-value of <0.01 were visualized as a network (Figure 5). In the PSS group (Figure 5a), *Paeniglutamicibacter* exhibited the highest abundance, with strong interactions among its members. The number of positive and negative correlations was relatively balanced, indicating a complex and multifaceted ecological network. These network structures are generally considered more stable (19). Although the ICL group was initially defined as healthy, its microbial network revealed antagonistic relationships among *Paeniglutamicibacter*, *Escherichia*, and *Ralstonia* (Figure 5b). Negative correlations were primarily observed between *Paeniglutamicibacter* and *Ralstonia* and between *Paeniglutamicibacter* and *Escherichia*, indicating key microbial interactions. These findings were consistent with the clustering results presented in the heatmap and hierarchical clustering (hclust) analysis. These findings suggest that in the ICL group, the microbial community dominated by *Paeniglutamicibacter* may have been affected by interactions involving *Ralstonia* and *Escherichia*.

**Figure 6:**
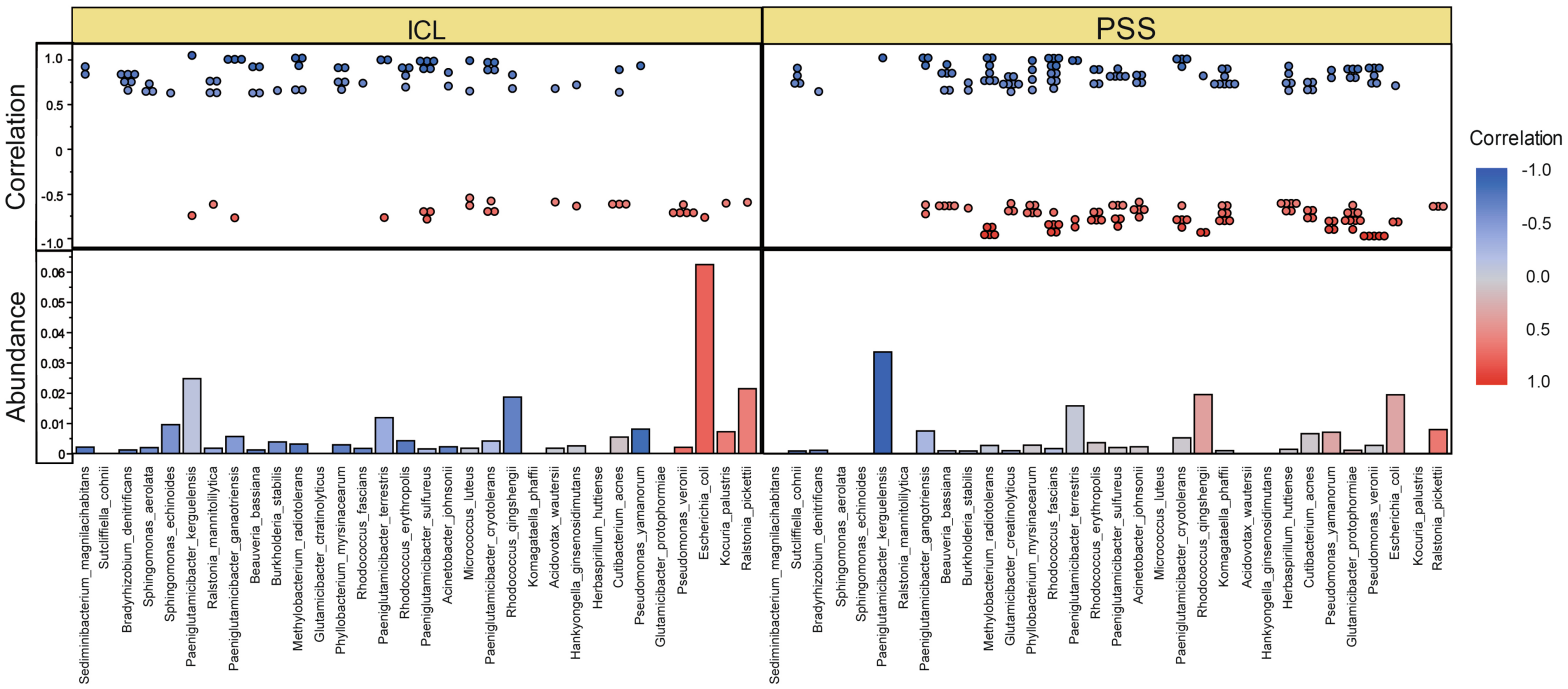
Comprehensive visualization of significant microbial correlations and abundance profiles. The upper dot plot illustrates pairwise Spearman correlations among the top filtered microbial species (retained based on presence in ≥30% of samples, |*r*| > 0.5, *p* < 0.001). Dot color represents correlation direction (red: negative; blue: positive), and color intensity reflects correlation strength. The lower bar chart displays the mean relative abundance of each corresponding microbial species in the ICL (blue) and PSS (red) groups, with the *Y*-axis on the right indicating the abundance scale. This integrated visualization highlights the predominance of negative correlations in the ICL group—particularly involving *Escherichia*, *Ralstonia*, and *Paeniglutamicibacter*—in contrast with the more balanced interaction patterns observed in the PSS group.

To quantitatively characterize the intricate relationships among these microorganisms, we constructed a comprehensive visualization integrating significant correlations and abundance profiles (Figure 6). The dot plot displays the strength and direction of all significant pairwise correlations (Spearman’s |*r*| > 0.5, *p* < 0.001) among the top filtered microbial species, with red indicating negative correlations and blue indicating positive correlations. The accompanying bar chart shows the mean relative abundance of each corresponding microbial species in the ICL and PSS groups, enabling a direct comparison between correlation patterns and group-specific enrichment. Consistent with the network analysis, this integrated visualization confirms the predominance of negative correlations in the ICL group, particularly involving *Escherichia*, *Ralstonia*, and *Paeniglutamicibacter*, while revealing more balanced interaction patterns in the PSS group.

### Predictive performance of microbial features for distinguishing PSS from ICL

**Figure 7:**
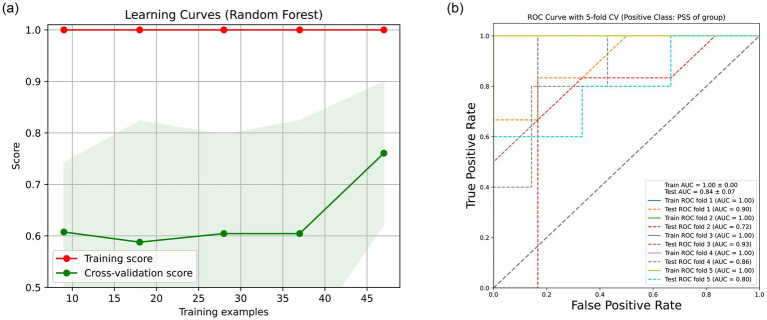
Predictive performance of aqueous humor microbial features for distinguishing PSS from ICL. **(a)** Learning curve of the random forest classifier. Model accuracy is plotted against the number of training samples. The curve demonstrates that accuracy increases with sample size, reaching 0.75 when 47 samples are used for training. **(b)** Receiver operating characteristic (ROC) curves from 5-fold cross-validation. Each gray line represents one fold, and the bold blue line shows the mean ROC curve. The mean area under the curve (AUC) is 0.84 (range: 0.7–0.9), indicating good discriminatory ability of the microbial features.

To assess the potential of the aqueous humor microbiome as a diagnostic tool, we evaluated the discriminatory performance of microbial composition using random forest classification. A learning curve was first constructed to examine the effect of sample size on model accuracy (Figure 7a). When the training set contained 9, 18, 28, and 37 samples, the average accuracy remained approximately 0.6. Increasing the training size to 47 samples raised the accuracy to 0.75, indicating that model performance is likely to improve further with larger cohorts. Using the full dataset with 5-fold cross-validation, the random forest model achieved a mean area under the curve (AUC) of 0.84 (range across folds: 0.7–0.9) for distinguishing PSS from ICL samples (Figure 7b). These results suggest that the microbial signatures identified in the aqueous humor hold promising potential as biomarkers; however, validation in independent, larger cohorts is required before clinical application.

## Discussion

In recent decades, the diversity and function of the microbiota associated with human health and disease have been extensively studied using high-throughput sequencing technologies and microbiomic/metagenomic analyses (20). However, the local microbiota of the eye under physiological and pathological conditions remains largely uncharacterized. Deng et al. (13) presented the first evidence of the existence of an intraocular microbiota in humans. The pathophysiology of PSS remains under debate, with the inflammation-related hypothesis gaining increasing attention (2). To deepen our understanding of the pathophysiology of PSS, we conducted a microbiome analysis integrating metagenomic sequencing data from both negative controls and AH samples with clinical information. mNGS technology, offering an unbiased approach, can detect not only bacteria but also fungi and viruses, capturing more complex microbial ecosystems (21). Our findings demonstrate, for the first time, the low microbial diversity, specific microbial enrichment patterns, and dynamic changes in the ecological interaction network within the AH of PSS patients, providing novel insights into the microbiome mechanisms underlying immune-related eye diseases. Random forest analysis identified key microorganisms associated with disease status (22), suggesting a potential role for the microbiota in the pathogenesis of PSS, and has laid the foundation for future functional gene analysis to explore microbial metabolism and immune regulation in PSS.

Compared to the ICL group, the PSS group showed a trend toward reduced microbial diversity; however, this difference did not reach statistical significance (Shannon index: *p* = 0.065; Simpson index: *p* = 0.066). In contrast, the bacterial community composition was significantly altered in the PSS group (PERMANOVA: *p* = 0.019), suggesting that compositional shifts, rather than diversity loss, may be more closely associated with disease status. This phenomenon aligns with the trend of microbial community simplification observed in various autoimmune diseases, such as rheumatoid arthritis and systemic lupus erythematosus (23–26). The reduced diversity may reflect the suppressive effects of a chronic inflammatory environment on microbial ecology or enhanced microbial clearance due to immune dysregulation (27, 28). The findings of this study are also similar to those for other eye diseases, such as glaucoma, dry eye disease, and keratitis (13, 29–31).

The observed trend toward decreased microbial diversity, although not statistically significant, is consistent with patterns reported in other ocular inflammatory diseases, as mentioned above, and may reflect underlying pathological mechanisms warranting further investigation. Notably, the trend toward reduced diversity appeared more pronounced in younger individuals (≤32 years) and male patients within the PSS group, raising the possibility that age and sex may influence microbiome stability through hormonal or immune regulatory pathways (32). However, given that subgroup analyses did not reach statistical significance, this observation remains speculative and requires validation in larger cohorts. Previous reports have shown that men are more at risk of the disease, with numbers varying from 50.5 to 71.4% (2, 33). Existing evidence also suggests a bidirectional relationship between microbiomes and sex hormones (34). The commensal microbial community is involved in sex steroid metabolism, which can influence hormone homeostasis and alter sex hormone levels, potentially affecting the development and prognosis of autoimmune diseases (35, 36). Differences in sex hormones may also contribute to sex bias in diversity in the microbial composition (37). In our study, we observed this sex bias in the intraocular microbiome, and further studies may confirm the interaction between the microbiome and sex hormones. PCoA further revealed significant intergroup microbial structural segregation (PERMANOVA *p* = 0.05), indicating that the disease state of PSS patients may be related to microbiome remodeling.

The LEfSe analysis revealed that the PSS group exhibited a high abundance of Actinobacteria, particularly *Paeniglutamicibacter* spp., which may play a role in the regulation of chronic inflammation. Actinobacteria, particularly *Paeniglutamicibacter*, are widely distributed in the environment, including soil and water (38, 39), and are known for their ability to degrade complex organic matter within ecosystems (40). Some members of *Actinobacteria* exhibit either symbiotic or pathogenic potential, particularly in immunocompromised individuals. For example, other actinobacteria, such as *Corynebacterium* species, have been extensively studied in immunosuppressed patients (41). Although *Paeniglutamicibacter* species are not recognized as major pathogens, they may become opportunistic pathogens under conditions of immunosuppression or impaired body barrier function. They may also produce anti-inflammatory metabolites, and the enrichment of this genus may represent an attempt by the host to balance the inflammatory response through microbial metabolism. However, excessive proliferation of these bacteria may also exacerbate autoimmune reactions via molecular mimicry mechanisms (42). Notably, the inverse relationship with IOP, specific to the PSS group, may reflect complex host–microbe interactions. Possible explanations include immunomodulatory effects of *Paeniglutamicibacter* on inflammation-mediated IOP elevation (43) or temporal variability in microbial load relative to disease flares. The absence of a correlation with medication use suggests that these associations are not confounded by treatment intensity. Prospective longitudinal studies capturing microbial dynamics across inflammation cycles are needed to elucidate causality and directionality.

In contrast, the ICL group, which served as the control group, exhibited enrichment of Proteobacteria, particularly *Escherichia coli*, which is often associated with gut microbiota translocation or environmental exposure (44). This unexpected enrichment of *E. coli* in the ICL group may be attributed to multiple factors, including transient bacterial migration from the gut due to physiological stress, perioperative antibiotic use, or contamination during sample collection. Additionally, *E. coli* colonization in the AH could reflect host–microbiota interactions influenced by ocular surface exposure or immune regulation. Notably, the high LDA score of *E. coli* in the ICL group (ranking first) and its significant enrichment in younger individuals suggest a potential age dependence of microbe–host interactions. This finding raises questions about whether age-related differences in immunity contribute to distinct microbial compositions in the AH. Further studies are needed to validate the relationship between *E. coli* enrichment and disease progression, as well as to clarify its potential role in ocular homeostasis or pathology.

Recent studies have expanded our understanding of the ocular microbiome beyond the conjunctival surface. Zhao et al. (45) demonstrated that the presence of an ocular prosthesis significantly alters conjunctival sac microbial diversity, whereas subsequent studies revealed dynamic shifts in ocular microbiota following blepharoplasty (46). These findings underscore the plasticity of the ocular surface microbiome and its responsiveness to both pathological conditions and surgical interventions. Collectively, these studies, together with our observations of distinct microbial signatures in the aqueous humor of PSS patients, suggest that the intraocular microbiome may be similarly dynamic. Further studies are needed to determine whether microbial alterations observed in PSS are drivers or consequences of the inflammatory process.

The multi-interaction network of the PSS group, characterized by a balanced mix of positive and negative correlations, aligns with the “high complexity–high stability” ecological theory (19). These findings suggest that despite reduced diversity, the remaining microbiota may maintain ecological balance through functional redundancy. In contrast, the antagonistic relationship (predominantly negative correlations) between *Paeniglutamicibacter, Ralstonia, and Escherichia* in the ICL group may reflect a competitive inhibition mechanism typical of microbial communities in a healthy state. This finding is consistent with the hclust clustering results, which showed that *Ralstonia* and *Escherichia* formed a distinct branch in the ICL group, potentially suppressing *Paeniglutamicibacter* colonization through resource competition (47). However, caution is needed when interpreting such phenomena in the ICL group as indicative of infections, because cross-sectional studies cannot establish causality. Furthermore, the origin of the AH microbiota (endogenous vs. exogenous) remains to be clarified through *in situ* hybridization or single-cell sequencing.

The observed dysbiosis in PSS, particularly the enrichment of *Paeniglutamicibacter* and the reorganized ecological network, strengthens our hypothesis regarding the potential interplay of the intraocular microbiome with the intraocular inflammatory environment. Previous studies have established that the AH levels of IL-1β, IL-5, IL-6, IL-8, IL-10, IFN-γ, and TNF-α were significantly elevated in PSS patients, which are crucial mediators of intraocular inflammation and can influence the breakdown of the blood–aqueous barrier (48, 49). Although our study did not directly measure these immune mediators, the distinct microbial community structure in PSS, characterized by a more interconnected and stable network, may reflect an adaptation to these chronic inflammatory environments or modulators of them. We hypothesize that the shift from the competitive, antagonistic microbial interactions observed in the control eyes to the more cooperative network in PSS could influence local immune homeostasis. The balanced positive-to-negative correlation ratio in the PSS network, as opposed to the predominantly antagonistic ICL network, may thus represent a microbial community under immune-mediated selective pressure, potentially contributing to the recurrent, relapsing–remitting nature of the disease. This speculative link between microbial ecology and the inflammatory cytokine network underscores the need for future integrated studies that concurrently profile both the microbiome and the metabolome/immunome in the same aqueous humor samples to unravel the directionality and mechanisms of these complex interactions.

## Limitations

This study has several limitations. First, the sample size was relatively small, and the sex imbalance in the PSS group (predominantly men) may affect the generalizability of the results. Although we performed multivariate analyses adjusting for age and sex, residual confounding cannot be entirely excluded, emphasizing the need for future studies with larger, well-matched cohorts or propensity score-matched designs to validate our findings and isolate disease-specific microbial signatures.

Second, the cross-sectional design made it difficult to infer causal relationships between microbial alterations and disease progression. The role of observed dysbiosis as a cause or consequence of the inflammatory process in PSS remains unclear.

Third, the functional characterization in this study relied on taxonomic associations and literature-based inferences; definitive functional validation was precluded by the scarcity of aqueous humor samples. This study included myopia patients as controls, which was necessary because of the challenge in obtaining samples from healthy eyes. Although this choice avoids the confounding effect of lenticular pathology in cataract patients, altered ocular physiology in myopia indicates that their baseline microbiome may not represent a fully healthy state, potentially confounding the results.

Fourth, contamination control is a critical concern in low-biomass microbiome studies. Although we incorporated negative controls (conjunctival sac irrigation fluid and BSS) and implemented stringent bioinformatic decontamination strategies, we acknowledge that additional controls, such as DNA extraction blanks and reagent-only controls, would further strengthen the rigor of contamination assessment.

Fifth, the AH samples were consumed during metagenomic sequencing and we were unable to perform targeted qPCR for key taxa or measure inflammatory markers to directly link microbial abundance with immune mediators. It is important to emphasize that metagenomic sequencing detects microbial DNA, which may originate from live organisms, dead cells, or environmental contamination. Therefore, our findings do not prove the presence of active, metabolically functional microorganisms, and the inferred immunological roles of *Paeniglutamicibacter* remain speculative. Despite the implementation of rigorous contamination controls, including the sequencing of conjunctival and BSS negative controls and the confirmation that the dominant taxa in our samples were distinct from those found in the controls, the low-biomass nature of aqueous humor remains a challenge. The possibility of undetected or stochastic contamination influencing our results, particularly for low-abundance taxa, cannot be entirely ruled out.

Additionally, differences in preoperative antibiotic protocols between the groups (longer duration in ICL vs. immediate preoperative dosing in PSS) and the use of corticosteroids in PSS patients may have differentially influenced the microbial communities. Although our correlation analyses showed no association between medication use and *Paeniglutamicibacter* abundance, the potential confounding effects of these treatment regimens cannot be fully excluded. We also acknowledge that functional characterization was not performed in this study. Owing to the low-biomass nature of aqueous humor samples, our sequencing depth was optimized for taxonomic profiling rather than reliable functional gene inference.

Further studies incorporating deep sequencing or metatranscriptomic approaches, larger sample sizes, and more extensive negative control strategies are warranted to validate our findings and to explore the functional potential of the intraocular microbiome.

## Conclusion

In conclusion, this exploratory study observed a trend toward reduced diversity in the aqueous humor microbiome of PSS patients, identified potential disease-associated microbial signatures, and revealed distinct ecological network patterns compared to controls. These findings provide preliminary evidence supporting the concept of intraocular microbiome dysbiosis in PSS. With appropriate caution, we suggest that these observations may provide a foundation for future investigations into microbiome-based biomarkers or therapeutic strategies for immune-related ocular diseases. We emphasize that further multicenter longitudinal studies, incorporating multi-omics approaches and rigorous experimental validation, are essential to elucidate the specific mechanisms of microbe–host interactions in the pathogenesis of PSS.

## Data Availability

The datasets presented in this study can be found in online repositories. The names of the repository/repositories and accession number(s) can be found at: https://www.ncbi.nlm.nih.gov/, PRJNA1155726.

## Footnotes

1
http://huttenhower.sph.harvard.edu/galaxy
