# Supplementary material for: Characterization of the aqueous humor microbiome in Posner–Schlossman syndrome: an exploratory metagenomic sequencing study
Source: Front Med (Lausanne). 2026 Apr 1;13:1780981. doi: 10.3389/fmed.2026.1780981 (PMC13079188; doi:10.3389/fmed.2026.1780981)
Supplement: Supplementary file 2 [file Table_2.docx]

**Table S2**. Demographic and ocular characteristics

| Parameters | PSS group (n=28) | ICL group (n=31) | P value |
| --- | --- | --- | --- |
| Gender (M/F) | 20/8 | 8/23 | 0.004 ^a^ |
| OD/OS | 12/16 | 19/12 | 0.196 ^a^ |
| Age (years, mean ± SD) | 41.21±12.58 | 29.39±6.52 | <0.001^b^ |
| BCVA (logMAR, mean ± SD) | 0.16±0.20 | 0.15±0.18 | 0.004 ^c^ |
| IOP (mmHg, mean ± SD) | 36.20±10.13 | 13.65±1.97 | <0.001 ^c^ |
| No. of medications (mean ± SD) | 2.59±1.01 | 0 | <0.001 ^c^ |
| C/D (mean ± SD) | 0.58±0.16 | 0.37±0.69 | <0.001 ^c^ |

PSS, Posner–Schlossman syndrome; HC, healthy control; M, male; F, female; OD, oculus dexter; OS, oculus sinister; SD, standard deviation; BCVA, best-corrected visual acuity; IOP, intraocular pressure; C/D, cup‒disc ratio; ^a^ Fisher's exact *t* test; ^b^ Unpaired *t* test; ^c^ Mann‒Whitney U test.
