# Supplementary material for: Characterization of the aqueous humor microbiome in Posner–Schlossman syndrome: an exploratory metagenomic sequencing study
Source: Front Med (Lausanne). 2026 Apr 1;13:1780981. doi: 10.3389/fmed.2026.1780981 (PMC13079188; doi:10.3389/fmed.2026.1780981)
Supplement: Supplementary file 3 [file Table_3.docx]

**Table S3.** The Fisher test results of the alternative grouping scheme.

|  | | | Ralstonia pickettii | Escherichia coli | Paeniglutamicibacter psychrophenolicus | All |
| --- | --- | --- | --- | --- | --- | --- |
| Gender | Female | Counts | 6 (24) | 4 (26) | 20 (10) | 30 |
|  | Male |  | 2 (22) | 4 (20) | 18 (6) | 24 |
|  | P value (Fisher’s exact test) | | 0.277 | 1 | 0.561 |  |
| Old | Middle | Counts | 2 (26) | 1 (28) | 25 (3） | 28 |
|  | Young |  | 6(23) | 7 (19) | 13 (13) | 26 |
|  | P value (Fisher’s exact test) | | 0.253 | 0.02* | 0.002* |  |
| Group | ICL | Counts | 6 (25) | 7 (24) | 18 (13) | 31 |
|  | PSS |  | 2 (21) | 1 (22) | 20 (1) | 23 |
|  | P value (Fisher’s exact test) | | 0.443 | 0.118 | 0.004* |  |
