# Supplementary material for: Characterization of the aqueous humor microbiome in Posner–Schlossman syndrome: an exploratory metagenomic sequencing study
Source: Front Med (Lausanne). 2026 Apr 1;13:1780981. doi: 10.3389/fmed.2026.1780981 (PMC13079188; doi:10.3389/fmed.2026.1780981)
Supplement: Supplementary file 4 [file Table_4.docx]

**Table S4.** Distribution of differential microorganisms between groups

|  | | | ***Ralstonia pickettii (R)*** | ***Escherichia coli (E)*** | ***Paeniglutamicibacter psychrophenolicus (P)*** | ***All*** |
| --- | --- | --- | --- | --- | --- | --- |
| **Gender** | Female | Counts | 7 (24) | 4 (28) | 20 (11) | 31 |
|  | Male |  | 2 (25) | 4 (23) | 21 (6) | 27 |
|  | P value (Fisher’s exact test) | | 0.157 | 0.772 | 0.089 |  |
| **Old** | Middle | Counts | 5 (24) | 1 (28) | 23 (6) | 29 |
|  | Young |  | 4 (25) | 7 (22) | 18 (11) | 29 |
|  | P value (Fisher’s exact test) | | 0.772 | **0.023*** | **0.018*** |  |
| **Group** | ICL | Counts | 6 (25) | 7 (24) | 18 (13) | 31 |
|  | PSS |  | 3 (24) | 1 (26) | 23 (4) | 27 |
|  | P value (Fisher’s exact test) | | 0.508 | **0.048*** | **0.003*** |  |
| **Shannon** | | Mean±SD | 3.42±0.24 | 2.68±0.54 | 2.46±0.43 | 2.64±0.54 |
|  |  | Pair | R vs. E, R vs. P | E vs. P | |  |
|  |  | P value (Dunn’s test) | **0.0001*** and **0.0296*** | 0.7997 | |  |
