# Supplementary figures and images for: Characterization of the aqueous humor microbiome in Posner–Schlossman syndrome: an exploratory metagenomic sequencing study
Source: Front Med (Lausanne). 2026 Apr 1;13:1780981. doi: 10.3389/fmed.2026.1780981 (PMC13079188; doi:10.3389/fmed.2026.1780981)

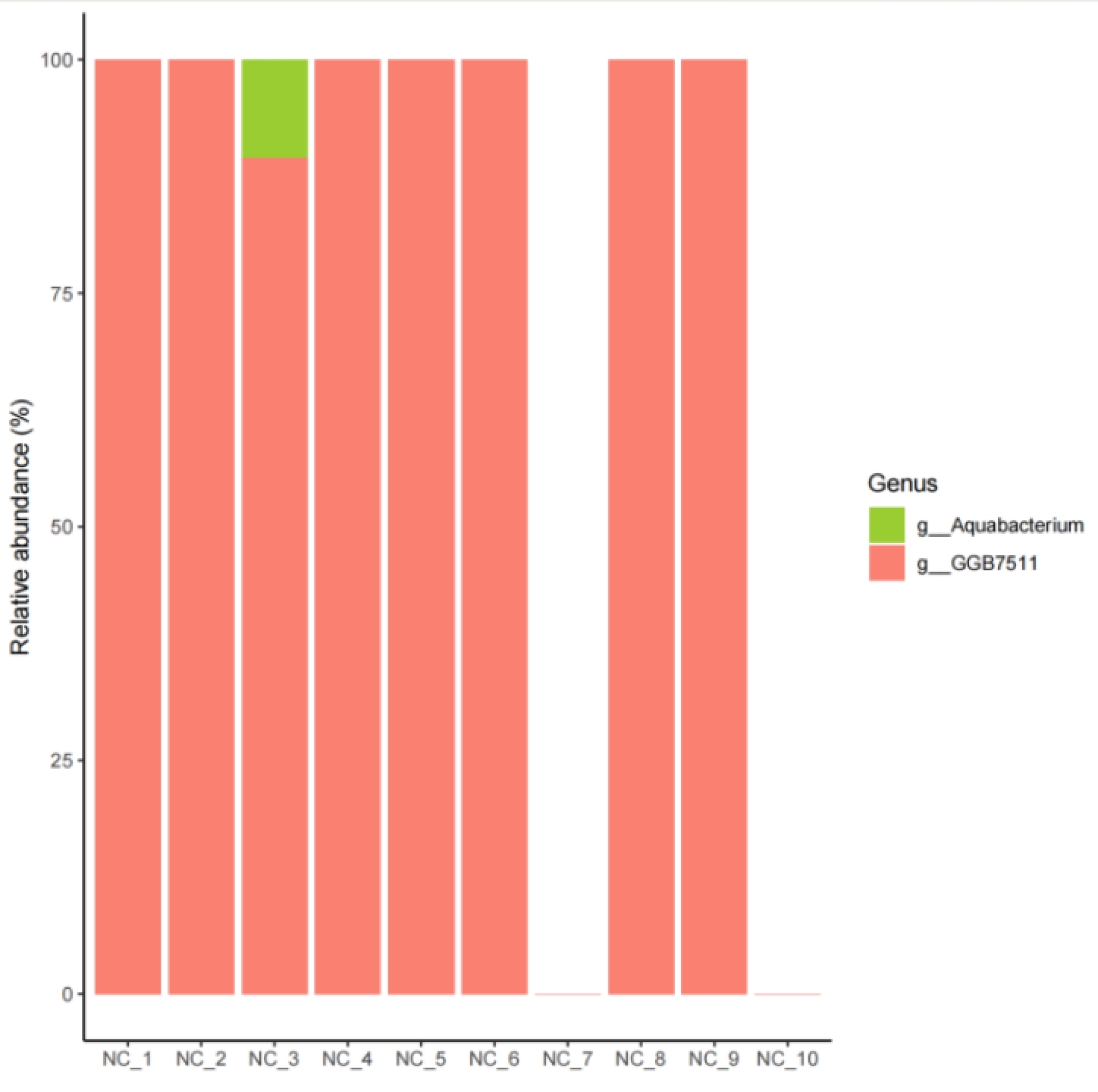

Supplement: Supplementary file 5 [file Image_1.jpeg]
